# Supplementary material for: Empirical methods for controlling false positives and estimating confidence in ChIP-Seq peaks
Source: BMC Bioinformatics. 2008 Dec 5;9:523. doi: 10.1186/1471-2105-9-523 (PMC2628906; doi:10.1186/1471-2105-9-523)
Supplement: Additional File 1 — A variety of html documents from the USeq web site detailing the available applications, their best usage, output file type descriptions, command line menus, etc. [file 1471-2105-9-523-S1.zip › USeqUserGuides/JavaDoc.zip!/JavaDoc/meme/package-summary.html]

meme


|  |  |  |
| --- | --- | --- |
| **Overview** \| **Package** \| Class \| **Use** \| **Tree** \| **Deprecated** \| **Index** \| **Help** | |  |
| **PREV PACKAGE**   **NEXT PACKAGE** | **FRAMES**    **NO FRAMES**     **All Classes** |  |


---

## Package meme

Wrapper for meme tweaked for processing multiple rounds of SELEX oligos.

**See:**
  
          **Description**

| **Class Summary** | |
| --- | --- |
| **MemeMotif** | This contains info generated from parsing a meme report, each found motif is represented by this class. |
| **MemeParser** | Parses meme output into MemeMotif objects and stores some generalize info re the meme run. |
| **MemeR** | Main, wRapper for meme geared toward processing multiple rounds of selex data. |
| **MemeResults** | Holds the results from various parts of MemeR. |
| **MotifHit** | Holds data related to a particular match of a matrix to a sequence. |
| **MotifScanner** | MotifScanner objects are used to scan sequences with a particular weight matrix and generate an array of MotifHit objects. |
| **MotifSearchResult** | Holds the results of searching a multi-fasta seq file with a particular motif. |
| **UtilMeme** | A catch all class to hold static methods and parameters set by different classes associated by MemeR. |

## Package meme Description

Wrapper for meme tweaked for processing multiple rounds of SELEX oligos.

---


|  |  |  |
| --- | --- | --- |
| **Overview** \| **Package** \| Class \| **Use** \| **Tree** \| **Deprecated** \| **Index** \| **Help** | |  |
| **PREV PACKAGE**   **NEXT PACKAGE** | **FRAMES**    **NO FRAMES**     **All Classes** |  |


---
